# The Coordination Chemistry of Bio-Relevant Ligands and Their Magnesium Complexes

**DOI:** 10.3390/molecules25143172

**Published:** 2020-07-11

**Authors:** Derek R. Case, Jon Zubieta, Robert P. Doyle

**Affiliations:** Department of Chemistry, Syracuse University, 111 College Place, Syracuse, NY 13244, USA; drcase@syr.edu

**Keywords:** magnesium, chelates, coordination chemistry, hypomagnesemia, nutraceuticals

## Abstract

The coordination chemistry of magnesium (Mg^2+^) was extensively explored. More recently; magnesium; which plays a role in over 80% of metabolic functions and governs over 350 enzymatic processes; is becoming increasingly linked to chronic disease—predominantly due to magnesium deficiency (hypomagnesemia). Supplemental dietary magnesium utilizing biorelevant chelate ligands is a proven method for counteracting hypomagnesemia. However, the coordination chemistry of such bio-relevant magnesium complexes is yet to be extensively explored or elucidated. It is the aim of this review to comprehensively describe what is currently known about common bio-relevant magnesium complexes from the perspective of coordination chemistry.

## 1. Introduction

Magnesium is the fourth most common element in the human body and is the second most common intracellular cation after potassium [1]. Magnesium is involved as a cofactor for more than 300 enzymes and 800 proteins [1,2,3] and it is required for energy production, oxidative phosphorylation, and glycolysis, amongst others. Magnesium contributes to the structural development of bone, with external layers of which aid in maintaining blood levels of magnesium [4] and magnesium deficiency linked to reduced bone mass [5,6]. Magnesium is required for the synthesis of DNA [7,8], RNA [9,10] and the antioxidant glutathione [11]. Magnesium also plays a role in the active transport of calcium and potassium ions across cell membranes, a process critical for nerve impulse conduction and muscle contraction including normal heart rhythm [12,13,14].

Several studies now support that an estimated 50–60% of Americans do not consume enough magnesium in their diets [15,16,17,18,19,20,21]. The concept of chronic latent magnesium depletion (hypomagnesaemia), defined as <0.75 mmol/L in blood [22] is not new, yet deficiency of magnesium remains an often unrecognized and widespread reality in the modern world [23]. Furthermore, insufficient magnesium is linked to a spectrum of clinical afflictions, including arrhythmias [24], hypertension [25,26], coronary artery disease [27,28,29], migraines/headaches [30], osteoporosis [31,32,33] and type 2 diabetes mellitus (T2DM) [28,34,35].

Given the prevalence of magnesium deficiency and hypomagnesaemia and its association with chronic disease states, there was a significant increase in interest in magnesium supplementation as a viable means of treatment [26,32,35,36]. This review focuses on the chemistry of bio-relevant magnesium complexes as it relates to complex geometry and degree of coordination, ligand coordination modes, behavior at varying pH values, inner- and outer-sphere coordination, and charge.

## 2. Discussion

### 2.1. Chemical/Physical Characteristics and Applications of Magnesium (Mg^2+^)

Magnesium was recognized as an element as early as 1755 and was isolated in 1808 [37]. Magnesium is a grayish metal [38], atomic number 12 [37], with an atomic weight of 24.3 Da [37].

Organomagnesium salts such as magnesium halides (of the type MgX; X = Cl, Br, etc.) are used extensively in Grignard-type organic syntheses [37,39]. Other organomagnesium complexes are biorelevant, such as magnesium citrate, and they are subject to constraints of the solution state. Magnesium has a high affinity for hydration water; more specifically, magnesium’s hydrated radius is approximately 400 times larger than that of its dehydrated radius [2]. In fact, it was previously established that, in aqueous conditions, not dissimilar to physiological conditions, magnesium will readily form hexaaquomagnesium ions ([Mg(H_2_O)_6_]^2+^) [40].

### 2.2. Biological Relevance of Magnesium

Evidence to support the biological relevance of magnesium is inscrutable. The importance of magnesium is placed heavily on the distribution of magnesium throughout the body and the processes through which it is utilized, such as the maintenance of bone mass and the synthesis of adenosine triphosphate (ATP). Subsequently, an understanding of magnesium distribution throughout the body is integral to understanding its biological relevance and implication as a coordinate cation [1,2,4].

#### 2.2.1. Storage and Occurrence

**Figure 1:**
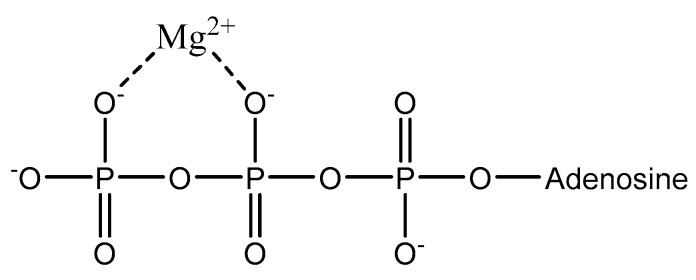
Structural illustration of the extracellular magnesium–ATP complex found in blood [1,3].

The average adult body contains ~25 g of magnesium [41]. Magnesium is stored unequally throughout the body; approximately 99% of bodily magnesium is intracellular with 90% stored in bone and muscle tissue, and the remaining < 1% is distributed in the blood [2,42,43,44]. Of the magnesium stored in bone and other assorted soft tissue, approximately 50–60% is stored solely within bone [1]. There is a delicate homeostasis maintained between bone concentrations and blood concentrations of magnesium, which results in the replenishing of blood magnesium from the outer layers of bone [4]. Magnesium within muscle and soft tissue accounts for 27% and 19% of total bodily magnesium, respectively [42]. The remaining 1% of bodily magnesium is distributed within the blood and is extracellular magnesium [2]. Extracellular magnesium exists as either free/ionized (55%), bound to proteins such as albumin or globulin (30%), or complexed with biological anions (15%) such as ATP (as illustrated in Figure 1) [1,2,45].

#### 2.2.2. Magnesium Uptake in the Gastrointestinal (GI) Tract

Uptake of magnesium thought the gastrointestinal tract (GI) varies, with the bulk of magnesium absorption occurring in the ileum (Table 1) [46].

Absorption throughout the GI is largely pH-dependent (Table 1), as illustrated by Thongon et al. [46,61], with the pH of the entire human small bowel ranging from 5.5–7 [47]. Increased magnesium absorption in the ileum is due to a more acidic luminal pH [48]. The unequal absorption of magnesium along the GI is also impacted by uptake pathway. The two available pathways that contribute to uptake are the non-saturable passive paracellular pathway (PPP), which is responsible for 80–90% of magnesium absorption along the length of the GI, and the saturable, active transcellular pathway (TP) [2,40,44].

The PPP is responsible for larger magnesium loads (≥250 mg) and is driven by an electrochemical gradient and solvent drag of Mg^2+^ between intestinal enterocytes. Successful propagation of Mg^2+^ between enterocytes is facilitated by tight-junction proteins known as claudins, which are responsible for stripping Mg^2+^ of its extensive hydration sphere [1,2]. The TP is responsible for the uptake of smaller magnesium loads (<125–250 mg); this is a result of the saturation of transient receptor potential channel melatstatin (TRPM 6 and TRPM 7), which facilitates removal of the magnesium hydration sphere, much like the claudins of the PPP [1,2,62,63]. At Mg^2+^ loads greater than those manageable by the TP, the bulk of uptake is mediated by the PPP [62,63].

### 2.3. Evaluating Magnesium Deficiency and Impact of Complex Composition on Uptake

Addressing magnesium deficiency status is complicated by several contributory factors. Firstly, 99% of total body Mg^2+^ is intracellular, with approximately 90% compartmentalized in bone and muscle, and the remainder (<1%) being distributed in blood [44]. This distribution of Mg^2+^ results in blood magnesium concentration being an inaccurate determinant of total body magnesium [5,27]. In addition, there are multiple complex factors that play a role in the absorption of magnesium such as utilization of both the TP and the PPP [1,2] and the impacts of intestinal luminal pH relative to magnesium absorption in the gut [22,64]. These factors make the claim of a certain percentage of gastrointestinal magnesium uptake, as based upon common analysis methods (Table 2), misleading [2]. Consequently, magnesium is one of the most poorly understood and appreciated minerals in human nutrition.

Nevertheless, understanding magnesium deficiency is integral to developing adequate treatment methods. Thus far, and most ubiquitously, magnesium supplements (including magnesium oxide, magnesium hydroxide, magnesium chloride, magnesium sulfate, magnesium lysinate, magnesium malate, magnesium glycinate, and magnesium aspartate) are used as a viable treatment for magnesium deficiency [26,32,35,36,65,66,67]. However, the physical properties of various Mg^2+^ forms will affect the behavior of any complex in the GI tract and beyond and will influence a key metric of any compound for oral ingestion, i.e., bioavailability.

### 2.4. Magnesium Bioavailability

There are many physical factors that were shown to impact magnesium bioavailability, such as dose load/frequency [68,69,70] and age [71]. There are also many chemical factors, most significantly solubility [67,72,73,74]. The bioavailability of Mg^2+^ within the GI exhibits correlative behavior relative to the water solubility of the complex. This indicates that complexes that exhibit greater solubility (see Table 3) within the physiological pH range of the GI tract are assumed to be more bioavailable.

Organic magnesium complexes show greater appreciable solubility than the commonly used magnesium oxide supplement, and the amino acids of these ligands occupy active sites on magnesium, reducing complexation with phytates and other inhibitory substances that reduce mineral absorption [1,2,46,62]. Occupation of these sites may reduce hydration of the magnesium, which could reduce the frequently encountered problem of laxation, a common feature with simple magnesium salts [1,2,46,62]. As such, organic- and amino-acid-based ligands as magnesium chelates provide an appropriate scaffold upon which to build and expand. However, given the factors that impact overall magnesium absorption from an administered complex, the design of an effective Mg^2+^ nutraceutical or pharmaceutical requires an appreciation of the chelation chemistry of magnesium and the subsequent coordinate ligand.

### 2.5. Biorelevant Organic and Amino Acid Magnesium Complexes and Their Coordination

**Figure 2:**
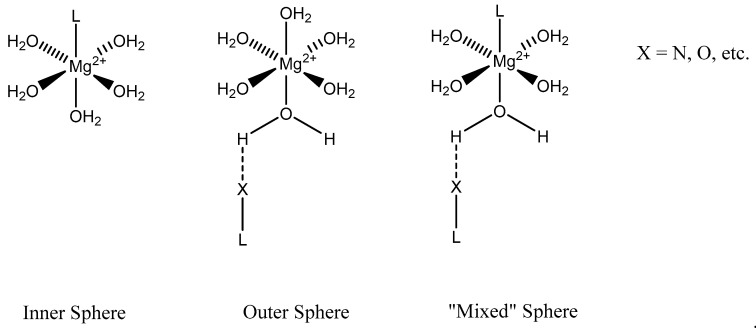
Illustration of the different ligand coordination modes utilized by magnesium. Adapted from Weston [40].

We focus primarily herein on biorelevant magnesium complexes of organic and amino acid ligands. Although evaluation of these interactions shows that ligand coordination can be both inner-sphere (magnesium glycinate) [82,83] and outer-sphere (magnesium orotate, utilizing a hexaquomagnesium core) [84], given magnesium’s high degree of hydration [2] (Figure 2), and interactions can be mono-, bi-, tri-, or tetradentate, little was done to determine the specific coordinative characteristics of specific magnesium–peptide complexes.

Organic and amino acid ligands that were previously evaluated regarding magnesium coordination are shown in Table 4. All pKa values were taken from the Chemical Rubber Company’s Handbook of Chemistry and Physics Table of Dissociation Constants of Organic Acids and Bases [85].

Understanding the pKa values of the terminal carboxylic acids, terminal amines, and available Lewis bases of the backbone of organic and amino acid chelates is integral to understanding the coordination chemistry of magnesium chelates. Changes in the pH of synthetic conditions can and will alter the outcome of the complex, as was the case with magnesium aspartate [65]. Whether or not an available Lewis base is protonated or deprotonated will dictate how a ligand coordinates, and, particularly in the case of amino acids with multiple Lewis bases, this may give rise to more entropically favored multidentate coordination modes.

#### 2.5.1. Orotic, Mandelic, and Anthranilic Acid

**Figure 3:**
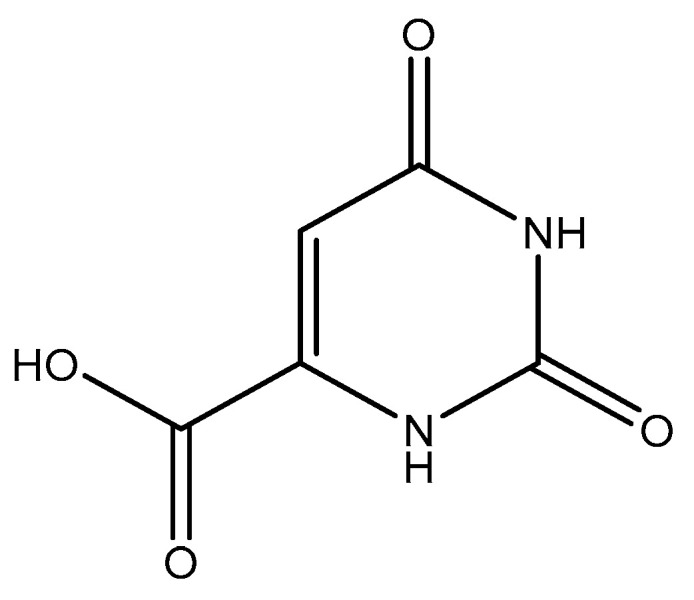
Structure of orotic acid.

**Figure 4:**
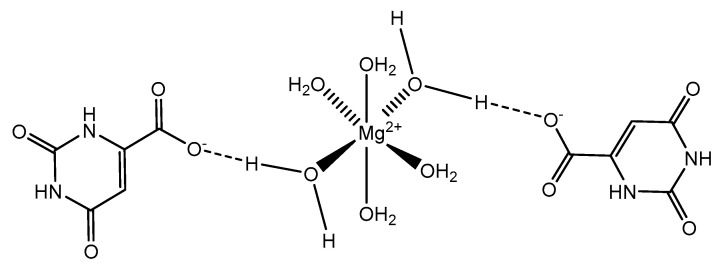
An illustration of the magnesium *bis*-orotate octahydrate reported by Schmidbaur et al. [84]. The pictorial representation is devoid of the two additional outer-sphere waters reported.

Orotic acid (6-uracilic acid, 1,2,3,6-tetrahydro-2,6-diox0-4-pyrimidinecarboxylic acid—Figure 3) [86,87] is a pyrimidine that is relevant to biological systems as a precursor molecule to pyrimidine nucleosides (e.g., uridine and cytidine) [88]. Given the established biological relevance of orotic acid, it presented itself as a viable option to generate a magnesium chelate that could be utilized to combat magnesium deficiency. Findings reported by Schmidbaur et al. showed that magnesium orotate and the analogous lithium complex could be synthesized in high yield via a simple neutralization of the appropriate hydroxide salt with two equivalents of orotic acid, with the product obtained as a colorless crystalline material [84]. Single crystal X-ray diffraction studies showed a magnesium *bis*-orotate octahydrate complex within the monoclinic space group P2_1_/C (Figure 4) [84].

As illustrated, the complexed orotic acid does not coordinate to magnesium as a traditional chelate. In fact, the orotic acid forms a complex with magnesium via outer-sphere coordination as a result of an extensive hydrogen bonding network—most notably, the interaction between the deprotonated terminal carboxylic acid and the ubiquitous hexaaquo magnesium core described by Weston [40]. Schmidbaur goes on to elaborate that there was no evidence to support that orotic acid would replace water within the inner sphere of the magnesium and subsequently stated that magnesium *bis*-orotate tetrahydrate, if bought commercially, would form the described octahydrate in aqueous solution at ambient temperature [84]. Similar findings were produced by Schmidbaur after the synthesis of magnesium *bis*-anthranalite dehydrate [89]. Schmidbaur further spoke to a similar form of outer-sphere coordination exhibited by citric acid as was initially described by Johnson et al. [90].

**Figure 5:**
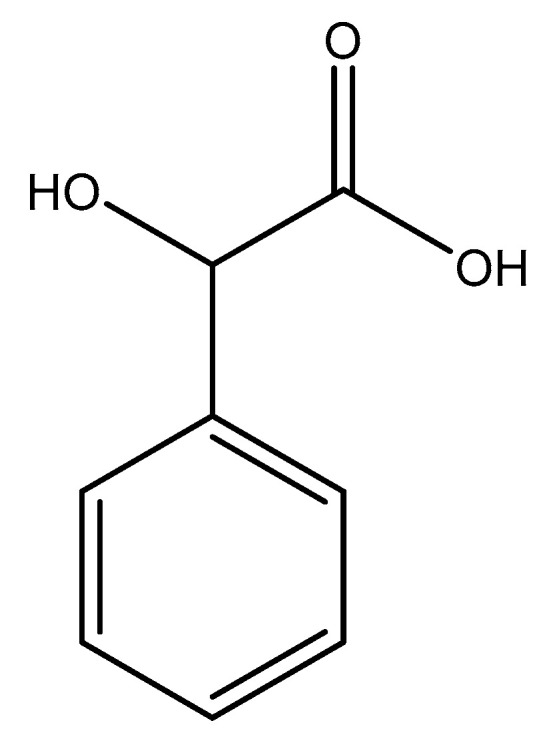
Illustrated structure of mandelic acid.

Schmidbaur went on to further evaluate the coordination of mandelic acid (mandelate—International Union of Pure and Applied Chemistry (IUPAC): hydroxyl (phenyl) acetic acid), a similar aromatic organic compound (Figure 5) [86,87] to magnesium. Given the structural similarities between orotic acid and mandelic acid, coordinative similarities are to be expected. However, there are four distinct differences between the two acids; the carboxylic acid side chain of mandelic acid is one carbon longer than that of orotic acid and contains a subsequent hydroxyl group, and the benzyl moiety of mandelic acid is devoid of any amines or carbonyls (structure at left).

**Figure 6:**
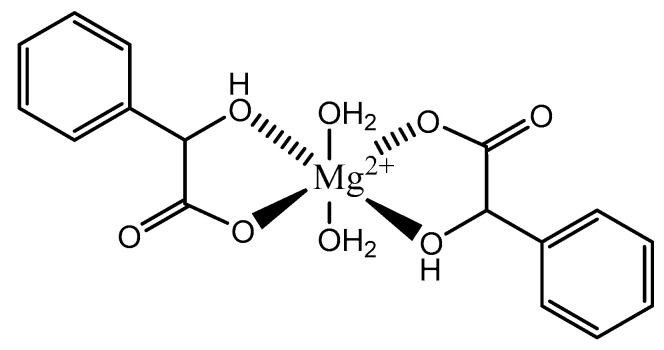
Illustrated structure of the magnesium *bis*-mandalate dihydrate structure proposed by Schmidbaur et al. [91].

Given the structure of mandelic acid, it is assumed that a magnesium complex formed with such a ligand would form a five-member ring structure and would require two mandelic acid ligands for charge balance as the magnesium is divalent and the mandelic acid ligand is only monoanionic. These assumptions coincide with the findings reported by Schmidbaur et al. (Figure 6) [91]

As shown, the mandelic acid ligand coordinates via an *O*,*O*-bidentate donor set and forms a stable five-member ring. The carboxylic acid of the mandelic acid becomes deprotonated, rendering it a carboxylate, and the backbone hydroxy remains protonated. Overall, the complex is six-coordinate and retains a strongly distorted octahedral geometry [91]. All coordination for the above complex is inner-sphere. Lastly, it should be noted that coordination of alcohol groups is not very common in aqueous magnesium chemistry [91].

**Figure 7:**
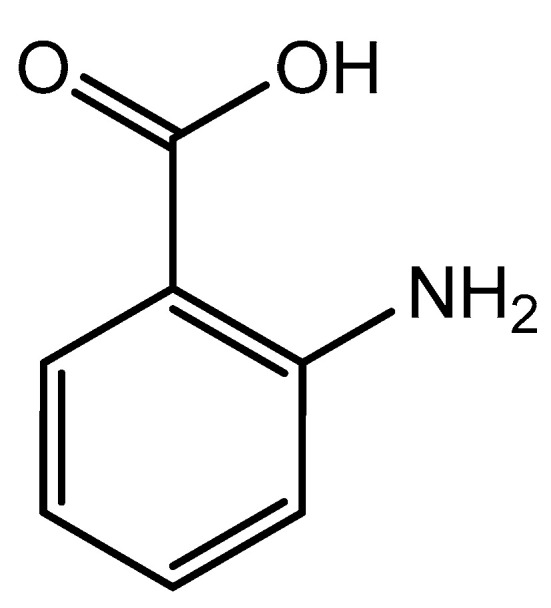
Structural illustration of anthranilic acid.

**Figure 8:**
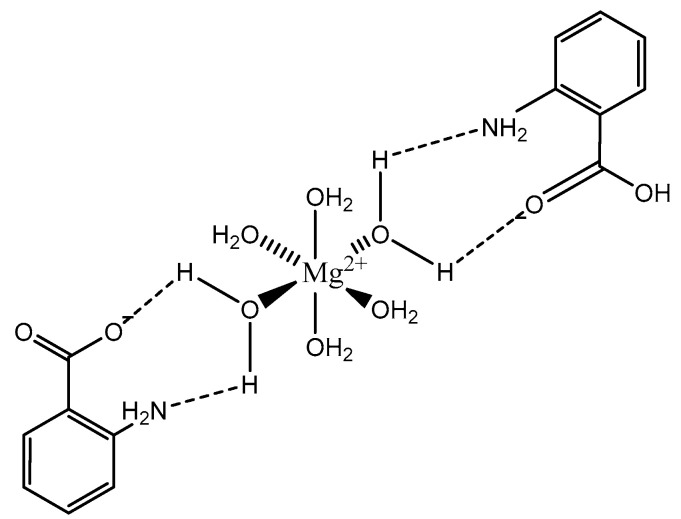
Structural illustration of the magnesium anthranilate structure proposed by Schmidbaur et al. [89]. The structure is devoid of two interstitial waters.

The coordination of anthranilic acid (IUPAC—2-aminobenzoic acid—Figure 7) [86,87] to magnesium was also investigated by Schmidbaur et al. [89]. Much like orotic acid, anthranilic acid does not act as a traditional magnesium chelate. In fact, the resulting magnesium anthranilate complex is the product of an extensive hydrogen bond network and is an octahydrate (Figure 8) [89]. As observed in the below illustration, all interactions between anthranilate and magnesium occur via outer-sphere coordination. The complex retains a hexaaquo magnesium core, and the anthranilate ligand coordinates via a series of hydrogen bonds between the amine and carboxylate group [89]. The complex retains a six-coordinate distorted octahedral geometry [89]. Schmidbaur noted that, when heated to 125 °C, the overall complex gave way to two other products—a hexahydrate that loses the interstitial waters, and an anhydrous product of only magnesium and anthranilate—but does not elaborate further as to the structure of these new species [89].

Of the aromatic magnesium structures studied by Schmidbaur, all three were octahedral, and two formed complexes solely through outer-sphere coordination via an extensive hydrogen bond that is common to magnesium complexes given the high propensity of magnesium to take on water in aqueous media [2,40].

#### 2.5.2. Formic Acid

**Figure 9:**
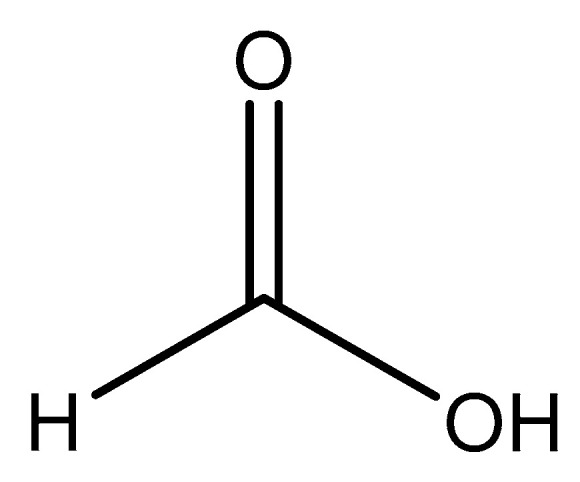
Structure illustration of formic acid.

**Figure 10:**
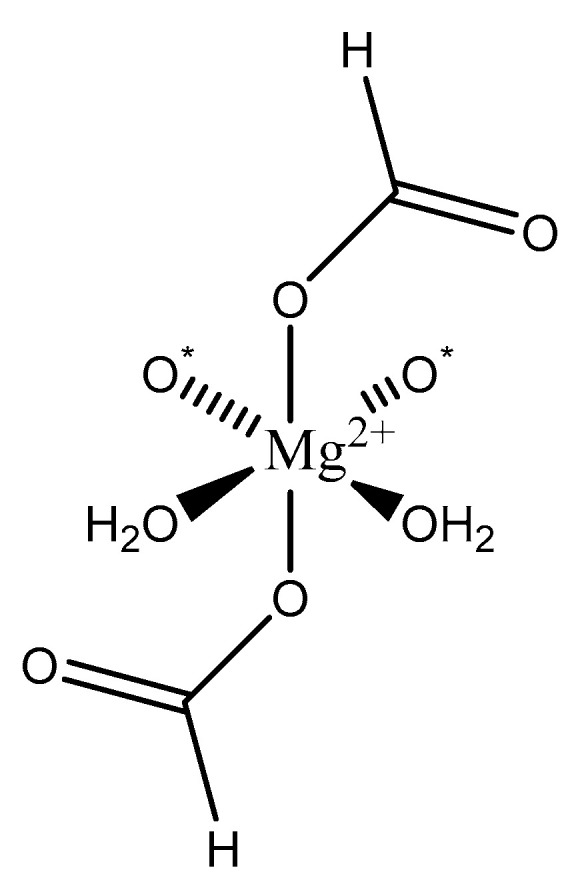
Illustrated structure of the magnesium *bis*-formate complex reported by Osaki et al. [98].

Formic acid (formate; IUPAC: methanoic acid—Figure 9) [86,87] is a naturally occurring carboxylic acid, and it is also the simplest carboxylic acid with the formula HCOOH. It is miscible with water and other polar organic solvents [92]. The conjugate base of formic acid, formate, is a common biproduct of the photo- and electrocatalytic reduction of carbon dioxide (CO_2_) to carbon dioxide (CO) [93,94,95,96]. Formic acid is readily derived from almost any plant biomass [97]. Formate is also used as a biomarker for methanol toxicity in the human body [92]. Magnesium formate structures were elucidated by Osaki et al. [98]. Since it only has one moiety that can be deprotonated, the formic acid conjugate base, formate, acts as a monoanion. It is likely the ligand will act as a monodentate ligand, but there is the possibly that it acts as a bidentate ligand given the resulting resonance structure that arises after deprotonation. The structure of magnesium formate described by Osaki et al. [98] is that of a magnesium *bis*-formate dihydrate (Figure 10).

In this case, the formate acts as a monoanionic monodentate ligand, which subsequently gives rise to the *bis*-formate chemical composition required for charge balance [98]. The remaining coordination sites are occupied by two waters and two oxygens from adjacent formate ligands [98]. The overall geometry of the complex is six-coordinate octahedral. Although the crystal structure in the solid state highlights two positions being occupied by oxygens from the surrounding formate ligands, undoubtedly, these two positions would be occupied by water in aqueous media, subsequently resulting in a tetrahydrate species rather than a dihydrate.

#### 2.5.3. Glycine

**Figure 11:**
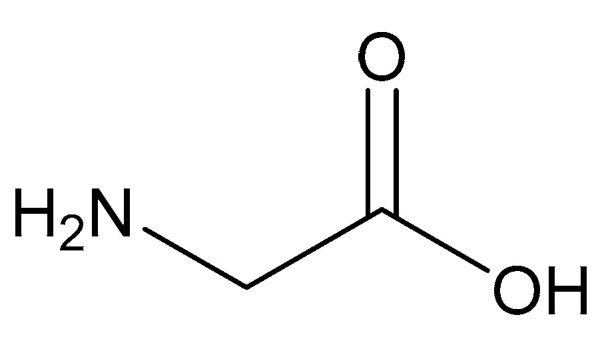
Structure of glycine.

**Figure 12:**
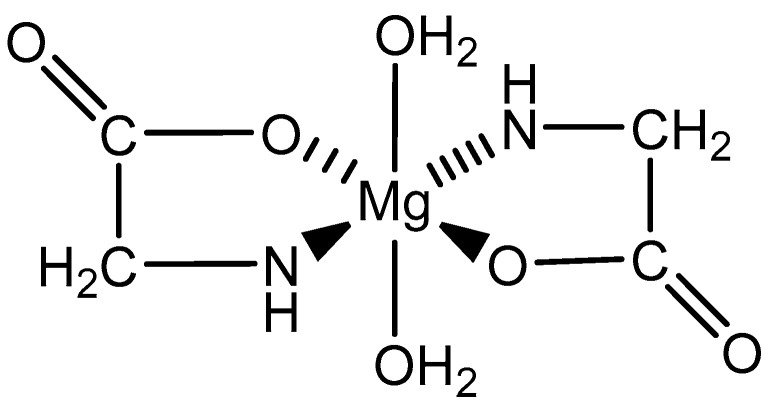
An illustration of the magnesium *bis*-glycinate dihydrate complex formed from a 1:2 magnesium–glycine stoichiometry [82,83].

As the simplest amino acid and the only amino acid without a stereocenter [99], glycine (IUPAC: 2-aminoacetic acid—Figure 11) [86,87] was an ideal starting point for the evaluation of biorelevant magnesium chelates. Initial studies to determine the coordination of glycine to magnesium at varying stoichiometries were conducted by Martell et al. [82,83]. Martell showed that, at a 1:1 stoichiometry of magnesium to ligand, only one ligand would coordinate to the magnesium center as a bidentate ligand, subsequently forming an entropically favored, highly stable five-member ring structure, with the remaining coordination sites being occupied by water and the overall complex retaining an octahedral geometry. At a 1:2 magnesium to ligand stoichiometry, it was shown that the magnesium–glycine complex would retain a magnesium *bis*-glycinate composition, again with the glycines acting as bidentate chelates and forming five-member rings, the remaining coordination sites being occupied by water, and the overall complex having an octahedral geometry (Figure 12). In both cases, the glycine ligand coordinated via inner-sphere coordination.

#### 2.5.4. Malic Acid and Maleic Acid

**Figure 13:**
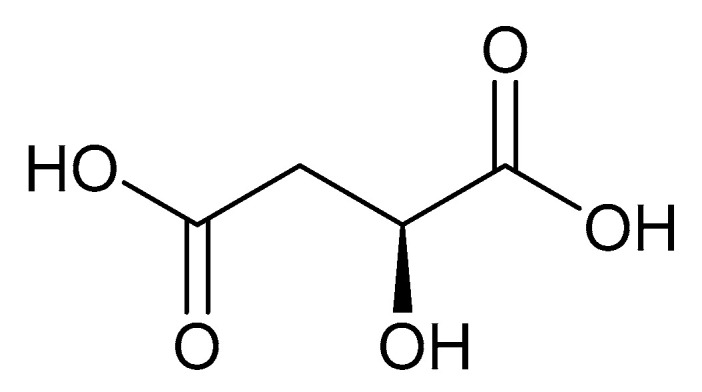
Structure of malic acid.

**Figure 14:**
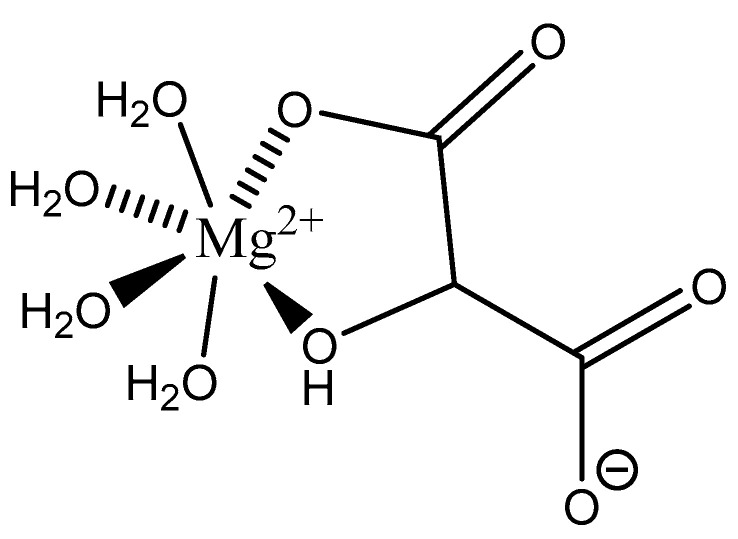
Structural illustration of the magnesium malic acid structure reported by Van Havere [102]. The structure is devoid of interstitial water.

Malic acid (2-hydroxybutanedioic acid—Figure 13) [86,87] is a dicarboxylic acid that exhibits substantial water solubility and readily impacts pH [100,101]. Given the inherent relationship between pH and magnesium absorption, malic acid provides the innate capability of buffering pH in regions of the small bowel where magnesium uptake is most substantial and, as such, is a useful magnesium chelate. Perusal of the literature indicates two described magnesium malate compositions and coordination modes based largely on reaction conditions, stoichiometry, and crystallization methods. One such structure, magnesium malate pentahydrate, was reported by Van Havere [102]. The malic acid acted as a true magnesium chelate (Figure 14)

As shown, the magnesium center assumes a six-coordinate, distorted octahedral geometry [102]. The malic acid ligand acts as a bidentate chelate and forms a highly stable five-member ring through one carboxylic acid and the backbone hydroxy group [102]. The remaining four coordination sites are occupied by water [102]. The resulting stoichiometry of the complex is 1:1 (M:L). No counterion is required for charge balance as the malic acid ligand is dianionic. In this case, all coordination is inner-sphere [102]. These findings are in conflict with findings reported in the early 1900s by Groth, who indicated the malic acid ligand acted as a tridentate ligand via an O_3_ donor set and the overall complex composition was that of a trihydrate [102].

**Figure 15:**
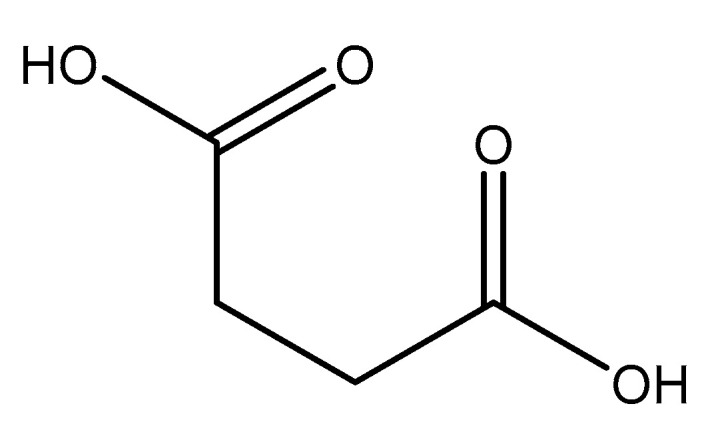
Structural illustration of maleic acid.

**Figure 16:**
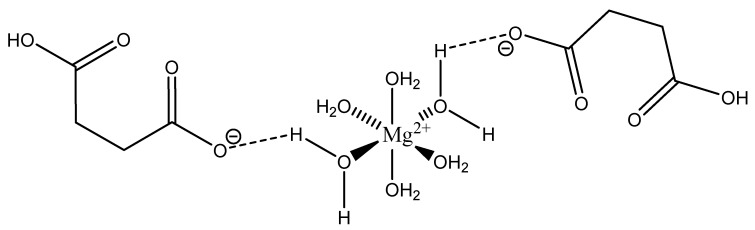
Structural Illustration of the magnesium *bis*(hydrogenmaleate) hexahydrate structure reported by Gupta et al. [103].

Maleic acid (IUPAC—*cis*-butenedioic acid—Figure 15) [86,87] is a dicarboxy acid like malic acid. A magnesium hydrogenmaleate structure was reported by Gupta et al. [103]. The structure showed a magnesium *bis*(hydrogenmalate) hexahydrate composition [103]. In this case, the maleate did not act as a traditional magnesium chelate, instead forming a magnesium complex via outer-sphere coordination to a hexaaquo magnesium core [103]. The hexaaquo magnesium core was six-coordinate and a near-perfect octahedron [103]. The hydrogen maleate coordinated to the hexaaquo magnesium core via a hydrogen bond between the deprotonated carboxylate group and one of the magnesium core waters [103]. The overall stoichiometry of the complex was 1:2 (M:L) and the resulting stoichiometry is what gives the complex overall charge balance, given the divalent magnesium and two maleic acid ligands acting monoanions. The overall structure is depicted in Figure 16.

It is most likely that this particular species, being complexed only via hydrogen bonding, would exist as the hexaquo magnesium core in aqueous solution.

#### 2.5.5. Aspartic Acid

**Figure 17:**
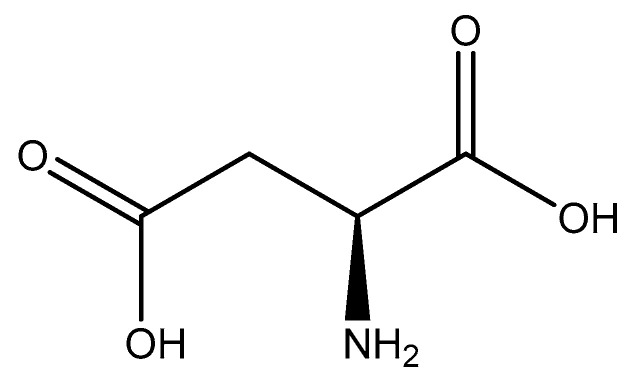
Structure of l-aspartic acid.

**Figure 18:**
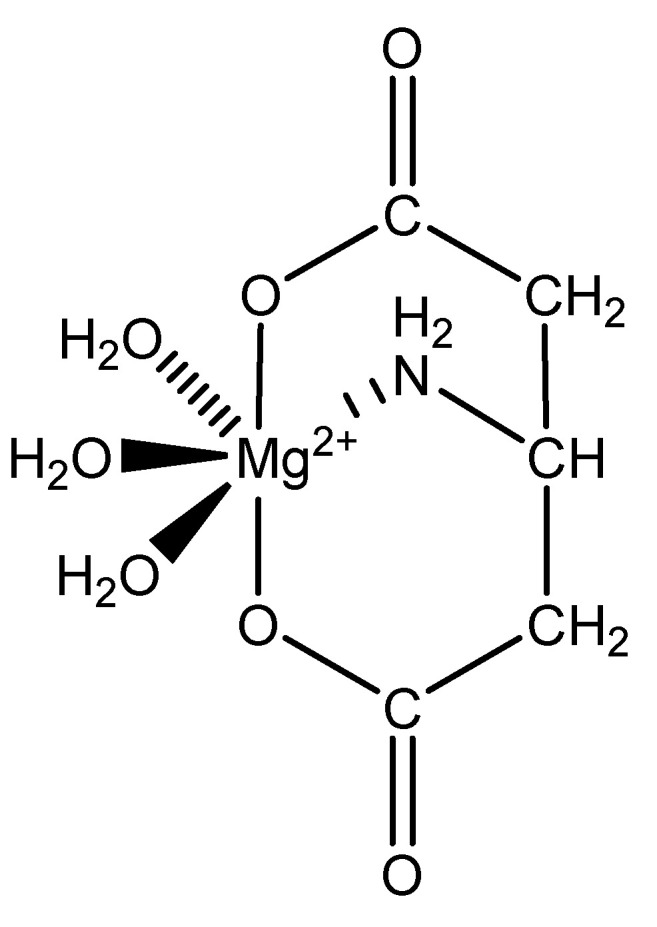
Illustrated structure of magnesium aspartate trihydrate [65].

Aspartic acid (aspartate; IUPAC: 2-aminobutanedioic acid—Figure 17) [86,87] is one of the 20 basic amino acids and is a nonessential amino acid, meaning that it is naturally produced by all mammals. Aspartic acid was first described by A. Plisson and E. Henry [104]. Aspartic acid is a dicarboxylic acid with substantial water solubility in aqueous solutions [105], and it has two stereoisomers: l-aspartic acid and d-aspartic acid; these isomers are a result of the changing stereochemistry of the backbone amine. Given their structural likeness, it comes as no surprise that aspartic acid also shows agonistic behavior at glutamate receptors [106]. Discussed at length before, the biorelevant orotic acid can also be synthesized from aspartic acid [107]. Given the biological applications of aspartic acid, it was evaluated as a magnesium chelate in the mid-1980s by Schmidbaur et al. [65]. From strongly alkaline solutions of magnesium aspartate (pH > 10), Schmidbaur determined that the aspartic acid ligand acted as a tridentate ligand with an N, O, O donor set via inner sphere coordination, and the overall magnesium aspartate complex was six-coordinate and retained a 1:1 magnesium to aspartate stoichiometry with octahedral geometry (Figure 18) [65,108].

**Figure 19:**
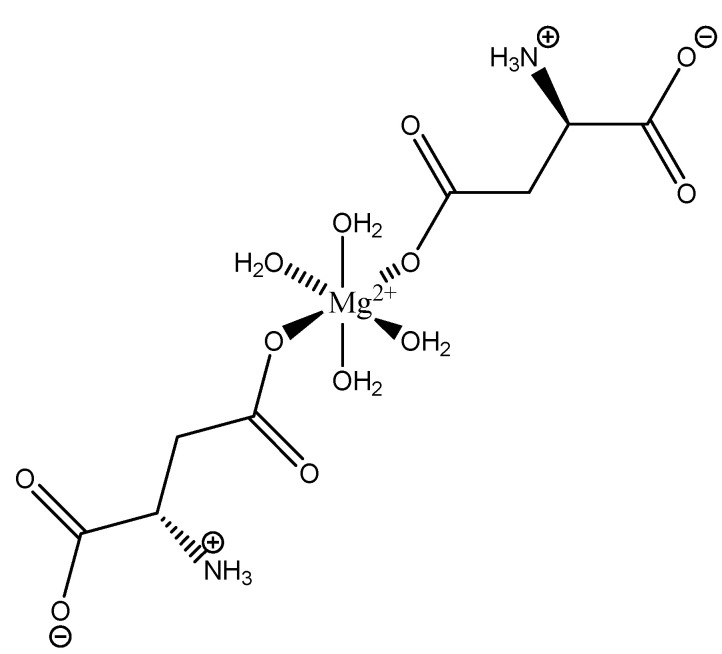
Illustrated structure of the magnesium *bis*(hydrogenaspartate) tetrahydrate structure reported by Schmidbaur et al. [65,109].

The inherent charge balance achieved by the above complex comes from the dianionic character of the aspartic acid. Further studies conducted by Schmidbaur et al. would indicate that the synthetic approach to magnesium aspartate complexes greatly impacts the outcome of isolated product. For example, Schmidbaur showed that a magnesium *bis*(hydrogenasparte) complex was generated in fair yield from the neutralization of magnesium oxide (MgO), magnesium hydroxide (Mg(OH)_2_), and magnesium carbonate (MgCO_3_) [65]. In this case, while both carboxylic acid moieties are deprotonated, the backbone amine remains protonated (-NH_3_^+^); this, in turn, alters the charge of the aspartic acid ligand from two-minus (−2) to one-minus (−1). This would result in the magnesium center requiring two aspartic acid ligands to achieve charge balance, and this occurrence was observed by Schmidbaur et al. (Figure 19) [65,109].

As illustrated, each aspartic acid ligand coordinates through one of the two available carboxylic acid moieties. The charge of the divalent magnesium is balance by the inherent one-minus (−1) charge of each hydrogen aspartate ligand. The one-minus charge of each ligand is the result of the pH of the synthetic conditions, which results in the protonation of the backbone amine. This protonation of the amine results in a positive charge (+1), and subsequently balances one of the negative charges (−1) from the two carboxylates, thus resulting in a net negative charge (−1) for each ligand [65,109]. The overall one-minus (−1) charge of the aspartate indicates that the divalent magnesium requires two aspartic acid ligands to achieve a charge-balanced complex, and an inherent 1:2 stoichiometry of the reported crystal structure supports this claim [65,109]. In this case, the aspartic acid ligand acts as a monodentate ligand, most likely the result of the backbone amine being protonated, in *trans* positions, and the overall complex is 6-coordinate with octahedral geometry [65,109]. The monodentate character of the aspartic acid ligand also speaks to the balance maintained between entropically favored bidentate complexes, and enthalpically favored complexes with a greater degree of hydration [65,109]. It should be further noted that the crystal structure clearly shows coordination of both the l- and d-aspartic acid isomers; this is accepted given the racemic mix of aspartic acid and the difficulty of isolating the enantiopure isomers [110].

**Figure 20:**
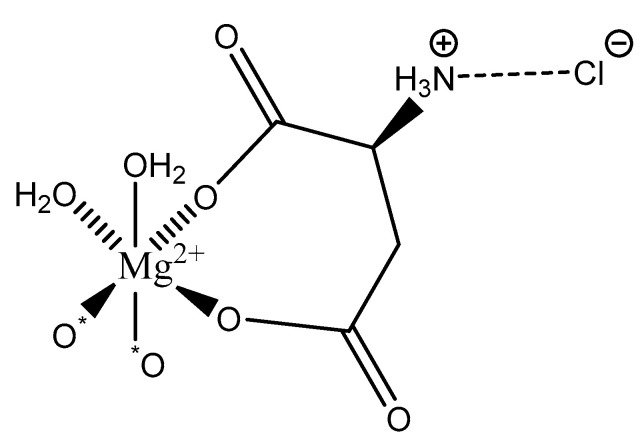
Illustrated structure of the Mg(l-Asp)Cl·3H_2_O reported by Schmidbaur et al. [65]. The structure is devoid of interstitial water that deems the overall complex formation a trihydrate.

Further studies conducted by Schmidbaur showed that the work-up of aqueous solutions containing Mg^2+^, AspH^−^, and Cl^−^ via spray-drying resulted in yet another magnesium aspartate complex of the composition Mg(l-Asp)Cl·3H_2_O (Figure 20) [65].

As illustrated, the structure is again six-coordinate octahedral. However, in this case, yet another coordination mode the aspartic acid ligand is observed: bidentate; the ligand coordinates via an O, O donor set from both the α- and β-carboxylic acids and forms an entropically favored seven-member ring. The backbone amine is precluded from magnesium coordination given its protonated state. Under reported synthetic conditions, both carboxylic acid moieties are deprotonated and the backbone amine is protonated [65]. While this should result in an overall one-minus (−1) charge of the ligand and subsequently the requirement of a *bis*(l-hydrogenaspartate) complex formation, the protonated amine is charge-balanced via hydrogen bonding to the chloride anion resulting in an overall dianionic net charge of the l-hydrogenasparate ligand and a 1:1 stoichiometry of magnesium:–l-hydrogenasparate [65]. Mg(l-Asp)Cl·3H_2_O retains a layered structure and an interstitial water molecule between each layer [65]. Two coordination sites of the magnesium subunit are occupied by water—resulting in an overall trihydrate composition—and the other two sites are occupied by carboxyl oxygens from neighboring units. It should be further noted that only the l-hydrogenaspartate ligand is observed, even though aspartic acid exists as a racemic mix.

Schmidbaur’s work with magnesium aspartate complexes illustrates the many available coordination modes of the dicarboxy ligand [65,109,111]. Schmidbaur’s collective works illustrate that the aspartic acid ligand, under varying synthetic approaches and pH ranges, will act as a mono-, bi-, and tridentate ligand [65,108,109]. However, regardless of the coordination mode of the ligand, the overall magnesium complex will assume six-coordinate octahedral geometry and achieve charge balance. Furthermore, resulting magnesium aspartate complexes are the product of a delicate balance between enthalpic and entropic terms [65].

#### 2.5.6. Glutamic Acid

**Figure 21:**
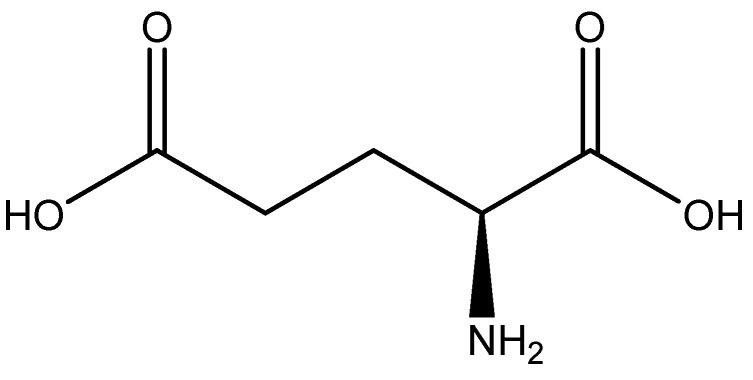
Structural illustration of l-glutamic acid.

Glutamic acid (glutamate; IUPAC: 2-aminopentanedioic acid—Figure 21) [86,87] differs from aspartic acid by a single methylene unit and is also a dicarboxylic acid with two stereoisomers (l-glutamate and d-glutamate) [112]. It was first discovered in 1866 by Ritthausen, and the chemical composition was later confirmed [113]. In addition to use in protein synthesis, glutamic acid biological is also a neurotransmitter, being responsible for mediating most of the fast excitatory neurotransmission in the central nervous system (CNS) [114]. Furthermore, approximately 80–90% of receptors in the brain are glutamatergic [114,115]. Glutamic acid is also a precursor for γ-aminobutyric acid (GABA) and is a constituent of glutathione [114]. As such, glutamic acid is of substantial biological importance, and it is an ideal candidate for magnesium coordination and subsequent supplementation.

**Figure 22:**
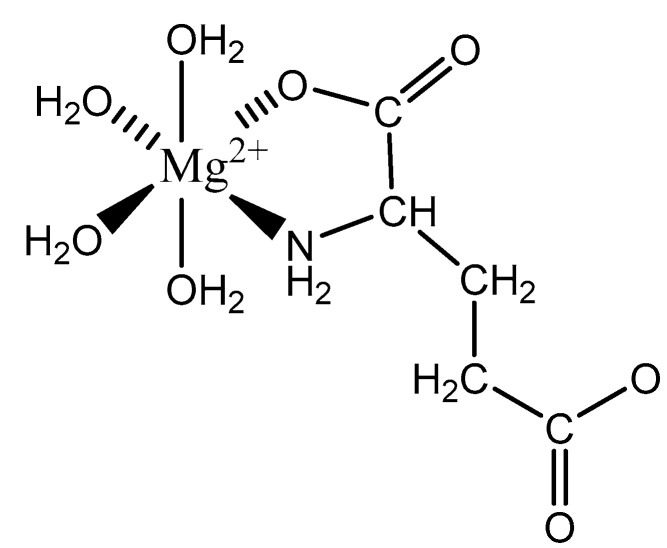
Structural illustration of Mg(l-Glu)·4H_2_O reported by Schmidbaur et al. [65,111].

Studies into the coordination of glutamic acid to magnesium were conducted by Schmidbaur et al. [65]. Schmidbaur reports that, from alkaline potassium hydroxide (KOH) solutions of magnesium glutamate, Mg(l-Glu) can be obtained as both a di- and tetrahydrate (2H_2_O, ·4H_2_O) [65,111]. However, Schmidbaur only reported on the crystallized structure of the tetrahydrate (Figure 22) [65,111].

As illustrated, the glutamic acid ligand coordinates as an *N,O*(α)-bidentate ligand via the α-carboxylate and the neighboring amine. In this case, the complex forms a five-member ring conformation with the glutamic acid ligand and chooses to forgo coordination to the γ-carboxylate; charge balance is still achieved due to the dianionic character of the glutamic acid ligand. This is interesting because aspartic acid and glutamic acid differ by only one carbon, and aspartic acid adopts a seven-member ring structure. It is likely the glutamic acid ligand adopts a five-member ring structure in favor of an eight-member ring structure due to differences in entropy—a claim supported by Schmidbaur [65,111]. The γ-carboxylate, while not participating in binding to the metal, does, however, participate in the extensive hydrogen bond network surrounding the complex in aqueous solution. In this case, the remaining magnesium coordination sites are occupied by four water molecules. The Mg(l-Glu)·4H_2_O retains a six-coordinate octahedral geometry and all coordination is inner-sphere. Schmidbaur was unsuccessful in crystallizing the dihydrate magnesium glutamate species, but it is likely that the reported species, with a higher degree of solvation, is most relevant in aqueous solution [65,111].

#### 2.5.7. Citric Acid

**Figure 23:**
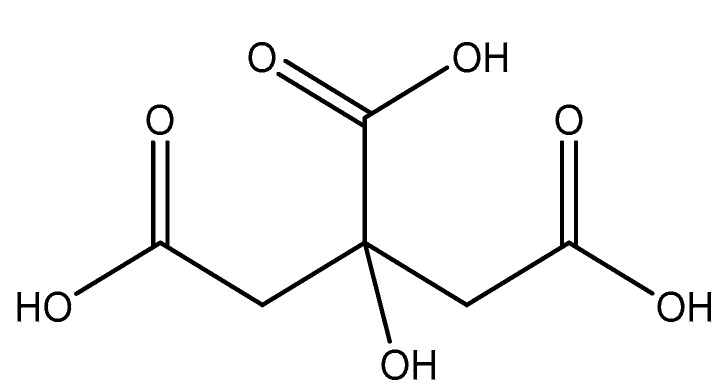
Illustrated structure of citric acid.

**Figure 24:**
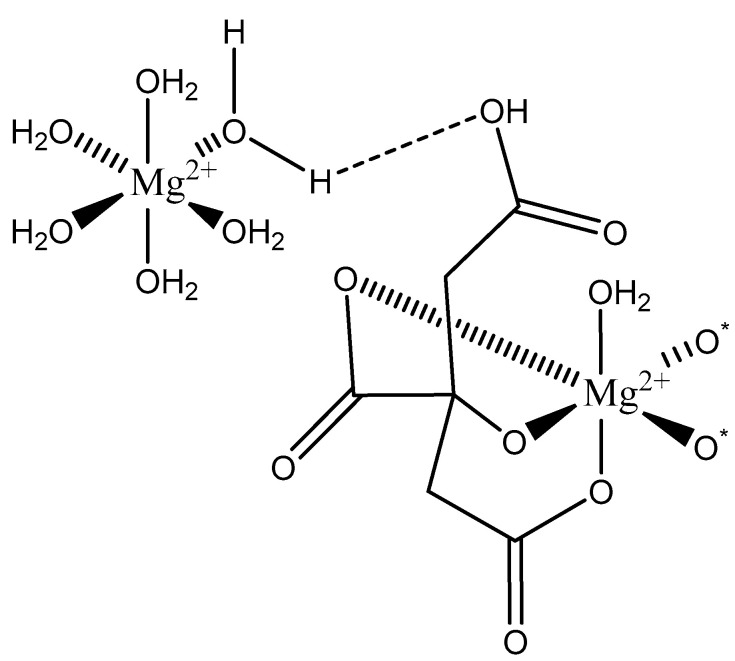
Illustrated structure representative of the structure reported by Johnson [90]. The structure does not illustrate interstitial waters. O* indicates contributed oxygen atoms from surrounding citrate molecules.

Citric Acid (citrate; IUPAC: 2-hydroxypropane-1,2,3-tricarboxylic acid—Figure 23) [86,87] is a tricarboxylic acid ligand with multiple Lewis bases. It is naturally occurring and was first isolated from lemon juice [116]. Perhaps most notably, citric acid is utilized during cellular respiration as part of the citric acid (Krebs) cycle [117]. Citric acid is used as a metal ion sequestrant and also shows appreciable water solubility [118]. As such, citric acid is a promising option as a magnesium coordinate ligand. Studies into the structure of magnesium citric acid complexes were reported on by Carroll K. Johnson in the early 1960s. Johnson reported on the structure of a magnesium citrate decahydrate complex (Figure 24) [90]; while a decahydrate may seem unreasonable, it is very reasonable given magnesium’s high propensity for hydration water and its extensive hydration sphere [2]. Highly solvated magnesium citrate complexes (a 14-hydrate) were also reported by Mansour et al. [119].

The structure reported by Johnson shows the crystal structure to contain two subunits (binuclear). The first subunit is that of a hexaaquo magnesium species (left), much like the species described by Weston [40], and a magnesium citrate species (right). In this case, the citrate does not participate in direct chelation to the magnesium at the center of the magnesium hexaaquo species, but does contribute a hydrogen bond [90] likened to that of outer-sphere coordination [40]. In the case of the magnesium at the center of the magnesium citrate complex, the citrate ligand coordinates via an O, O, O-tridentate donor set; all coordination is inner-sphere. The citrate forms an entropically favored six-member ring via one terminal carboxylate group and the central hydroxy group and a second entropically favored five-member ring via the central hydroxy group and the central carboxylate group [90]. It seems peculiar that the citric acid ligand would forgo the symmetry of two six-membered rings, but it stands to reason given the tendency of magnesium to form stable five-member ring structures with “hard” base donors such as that of magnesium *bis*-glycinate [82]. Further evaluation of the structure also indicates that both magnesium centers are six-coordinate and retain octahedral geometry; all coordinate positions of the hexaaquo magnesium are occupied by waters, and the positions of the magnesium citrate complex that are not occupied by the citric acid ligand are occupied by one water and two oxygens from the carboxylates of neighboring citrate molecules. The two metal centers of the crystal structure indicate a linkage via hydrogen bonding between the non-coordinated carboxylic acid of the citric acid ligand and one of the waters from the hexaaquo magnesium species.

## 3. Summary

The coordination chemistry of biorelevant magnesium ligands is a sparse field. Of the 20 basic amino acids, only four were reported as magnesium chelate ligands and subsequently elucidated structurally; only a small percentage of the possibilities of biorelevant magnesium complexes were revealed or utilized. In general, biorelevant magnesium complexes will assume a six-coordinate octahedral geometry and will almost always employ “hard” acids (N, O, etc.) as donors from the coordinate ligands. Coordinate ligands can assume many coordinate modes (mono-, bi-, or tridentate), and may be inner- or outer-sphere. For structures already solved, findings are reported in Table 5.

The coordination of biorelevant ligands to magnesium is altered by slight changes in many variables; these variables include protonated states of the ligand as a result of pH, the flexibility of the ligand, organic or aqueous media, and whether the entropy of a multidentate coordination of the ligands is favored over enthalpy. As is the case with many of the described complexes produced in aqueous media, coordination of the ligand occurs as a series of hydrogen bonds to hexaaquo magnesium core, suggesting that hydration of magnesium and the subsequent enthalpy is favored. However, in other cases, if the ligand has the capability to form stable five-, six-, or seven-member rings, a complex may be produced with the ligand acting as a bi-, tri-, or tetradentate ligand that is more entropically favored. These variables can be controlled synthetically. In addition, software was developed, which is effective at predicting complexes of these types of reactions. MINTEQ^TM^ is an open-source software platform that determines speciation based upon reaction conditions such as temperature, pH, ionic strength, and stoichiometries during synthesis, based in large part on speciation experiments conducted by Martell et al. [82,121]. Such programs are an invaluable resource for understanding biorelevant magnesium complex coordination chemistry in aqueous media.

## Figures and Tables

**Table 1:** Contribution of specific portions of the gastrointestinal tract (GI) on magnesium uptake.

GI Tract Segment	pH	Contribution of Magnesium Uptake (%)	Reference
Duodenum	5.9–6.8	11	[46,47,48,49,50,51,52,53,54,55,56,57,58,59,60]
Colon	5.7–7.2	11	[46,47,48,49,50,51,52,53,54,55,56,57,58,59,60]
Jejunum	5.9–6.8	22	[46,47,48,49,50,51,52,53,54,55,56,57,58,59,60]
Ileum	7.3–7.6	56	[46,47,48,49,50,51,52,53,54,55,56,57,58,59,60]

**Table 2:** Common methods used for the determination of magnesium status with pros and cons.

Analysis Method	Advantages	Difficulties
Serum	Rapid and easy	Does not reflect total body magnesium
Urine Excretion	Valuable for tracking kidney wasting (high [Mg^2+^]) or intake issues (low [Mg^2+^])	Time consuming
Isotopic Analysis	Confirmed role of small intestine in absorption	Confined to laboratory research

**Table 3:** Common magnesium supplements and their solubilities. Table includes pKa values for the terminal carboxylic proton (pKa_1_) and the amino proton (pKa_2_). Backbone protons are displayed as pKa_3_. The asterisk (*) represents the pKa of the first and second protons to be lost from the diacid sulfuric acid and the subsequent anion bisulfate, and the double asterisk (**) represents the pKa of the proton farthest from the backbone alcohol of the dicarboxylic acid malic acid.

Form (Oxide/Salt/Chelate)	Acid/Base Chemistry	Solubility (g/100 mL H_2_O)	MW g/mol (%Mg)	Reference
Magnesium oxide	Alkaline	0.010	40.30 (60.3)	[75]
Magnesium citrate	Acidic (pKa_1_ = 3.13)	20	214.41 (11.3)	[37]
Magnesium chloride	Neutral	56.0	95.21 (25.5)	[76]
Magnesium sulfate	Acidic (pKa_1_ = 3.0; pKa_2_ = 1.99 *)	35.7	120.37 (20.1)	[75]
Magnesium orotate	Acidic (pKa_1_ = 2.83)	Slightly soluble	334.48 (7.2)	[66]
Magnesium taurate	Acidic (pKa_1_ = 1.50)	Slightly soluble	272.57 (8.9)	[77]
Magnesium aspartate	Acidic (pKa_1_ = 1.88; pKa_2_ = 9.60; pKa_3_ = 3.65)	4.0	288.49 (8.5)	[78]
Magnesium threonate	Acidic (pKa_1_ = 3.4)	Soluble	294.50 (8.3)	[79]
Magnesium malate	Acidic (pKa_1_ = 3.46; pKa_2_ = 5.10 **)	Slightly soluble	156.37 (15.5)	[80,81]
Magnesium hydroxide	Alkaline	0.00069	58.32 (41.7)	[75]
Magnesium carbonate	Weakly alkaline	0.18	84.31 (28.8)	[37]

**Table 4:** Previously establishedand commonly used organic and amino acid chelate ligands for magnesium complexes. Table includes pKa values for the terminal carboxylic proton (pKa_1_) and the amino proton (pKa_2_). Backbone protons are displayed as pKa_3_. The asterisk (*) represents the secondary pKa of citric acid and malic acid, which have no amino proton—in the case of malic acid, the value is for the terminal carboxylic acid farthest from the backbone alcohol. The hyphen (---) indicates that the specified value does not apply to this specific ligand.

Ligand	pKa_1_	pKa_2_	pKa_3_	Lewis Bases
Orotic Acid 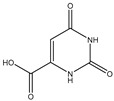	2.83	---	---	1
Mandelic Acid 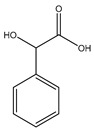	3.41	---	---	2
Anthranilic Acid 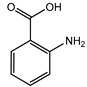	2.14	4.85	---	2
Formic Acid 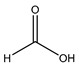	3.75	---	---	1
Glycine 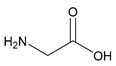	2.34	9.60	---	2
Malic Acid 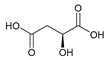	3.40	5.11 *	---	3
Maleic Acid 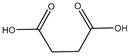	1.83	---	---	2
Aspartic Acid 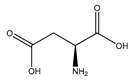	1.88	9.60	---	3
Glutamic Acid 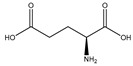	2.19	9.67	---	3
Citric Acid 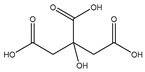	3.13	4.76 *	6.40	4

**Table 5:** Complete summary of all reviewed complexes including ligand coordination modes, complex coordination, and complex geometry. This table does not include those complexes reported by Yang et al. Yang supplies a full summary in his initial work [120]. The asterisk (*) denotes a shorthand to define complex geometry as either octahedral (O), distorted octahedral (D.O.), pseudo-octahedral (P.O.), tetrahedral (T), distorted tetrahedral (D.T.), or square planar (S.P.). The pound (#) indicates the coordination number of the described magnesium complex (i.e., the species is six-coordinate).

Complex	Ligand Coordination	Complex Coordination (#)	Geometry *	Reference
Orotic Acid 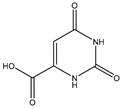	*(bis)* Monodentate (O)	Outer sphere (6)	D.O.	[84]
Mandelic Acid 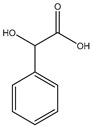	*(bis)* Bidentate (O,O)	Inner sphere (6)	D.O.	[91]
Anthranilic Acid 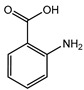	*(bis)* Bidentate (O,N)	Outer sphere (6)	D.O.	[89]
Formic Acid 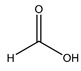	*(bis)* Monodentate (O)	Inner sphere (6)	O	[98]
Glycine 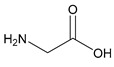	*(mono,bis)* Bidentate (O,N)	Inner sphere (6)	O	[82]
Malic Acid 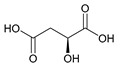	*(mono)* bidentate (O,O)	Inner sphere (6)	D.O.	[102]
H-Maleic Acid 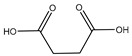	*(bis)* monodentate (O)	Outer sphere (6)	O	[103]
Aspartic Acid 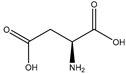	*(bis)* monodentate (O) *(mono)* bidentate (O,O) *(mono)* tridentate (N,O,O)	Inner sphere (6) Inner sphere (6) Inner sphere (6)	O O O	[65,109]
Glutamic Acid 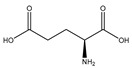	*(mono)* bidentate (O,O)	Inner sphere (6)	O	[65]
Citric Acid 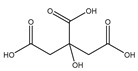	*(mono)* tridentate (O,O,O)	Inner sphere (6)	O	[90]
